# The Importance of the Circadian Clock in Regulating Plant Metabolism

**DOI:** 10.3390/ijms18122680

**Published:** 2017-12-11

**Authors:** Jin A Kim, Hyun-Soon Kim, Seo-Hwa Choi, Ji-Young Jang, Mi-Jeong Jeong, Soo In Lee

**Affiliations:** 1 National Academy of Agricultural Science, Rural Development Administration, 370, Nongsaengmyeong-ro, Wansan-gu, Jeonju-si, Jeollabuk-do 560-500, Korea; seohwa5009@naver.com (S.-H.C.); center1097@korea.kr (M.-J.J.); silee@korea.kr (S.I.L.); 2 Plant System Engineering Research Center, Korea Research Institute of Bioscience and Biotechnology, 125 Gwahak-ro, Yuseong-gu, Daejeon 34141, Korea; junging@kribb.re.kr

**Keywords:** carbohydrate, circadian clock gene, circadian rhythms, crop productivity, diurnal regulation, metabolism, photoperiodic control

## Abstract

Carbohydrates are the primary energy source for plant development. Plants synthesize sucrose in source organs and transport them to sink organs during plant growth. This metabolism is sensitive to environmental changes in light quantity, quality, and photoperiod. In the daytime, the synthesis of sucrose and starch accumulates, and starch is degraded at nighttime. The circadian clock genes provide plants with information on the daily environmental changes and directly control many developmental processes, which are related to the path of primary metabolites throughout the life cycle. The circadian clock mechanism and processes of metabolism controlled by the circadian rhythm were studied in the model plant Arabidopsis and in the crops potato and rice. However, the translation of molecular mechanisms obtained from studies of model plants to crop plants is still difficult. Crop plants have specific organs such as edible seed and tuber that increase the size or accumulate valuable metabolites by harvestable metabolic components. Human consumers are interested in the regulation and promotion of these agriculturally significant crops. Circadian clock manipulation may suggest various strategies for the increased productivity of food crops through using environmental signal or overcoming environmental stress.

## 1. Introduction

Plants are exposed to a daily alternation between light and dark with periods of approximately 24 h [1,2]. The rhythmicity of this day-night cycle gives the time information of environmental changes to plants fossilized throughout their life. Plants can measure time and predict coming change through their endogenous clock entrained to environmental time cues [3]. These Circadian rhythms, endogenous rhythms with periods of 24 h driven by an internal circadian clock, cause a variety of changes, including changes in transcription and post-transcriptional regulation in plants. The rhythms in stomatal conductance were described by Francis Darwin almost 100 years ago [4]. Circadian clocks were revealed to be composed of the products of genes in the 1970s. Clock genes can transcribe with free-running period [5]. The circadian clock genes play important roles in plants and account for one-third of Arabidopsis transcripts [6]. They are involved in numerous processes such as internal metabolic and hormonal signals, ranging from the control of metabolism, growth, development, and stomatal opening to metabolic processes [2,7]. Understanding how the circadian oscillator regulates these biological processes and affects productivity is an important agronomic issue.

Matching the endogenous clock period with the period of exogenous light-dark (LD) cycles provides an advantage by optimizing the phase relation between clock-controlled biology and exogenous day-night cycles. Correct matching of the circadian period with chlorophyll accumulation, CO_2_ fixation, and photosynthesis in the external period may increase vegetative growth and ultimately enhance productivity of crops [8,9]. In this review, we discuss the control of carbon assimilation and allocation by circadian clock genes in attempts to increase crop productivity.

## 2. Circadian Oscillation of Primary (Carbon) Metabolites

Many genes related to the photosystem, photosynthesis, and various key secondary metabolite pathways oscillate in the normal LD cycle [10]. In studies of transcriptomics and metabolomics in Arabidopsis, the circadian clock has been shown to regulate the transcript amounts of numerous enzymes involved in plant primary metabolism [10] and 30% of primary metabolite accumulation is under circadian control in normal growth conditions at 20 °C [11,12]. In plants, carbohydrates are potential energy sources, which are used in many cell synthesis reactions, such as the synthesis of proteins and lipids. Therefore, the carbon economy of the entire plant is important when investigating methods to increase crop productivity.

Mature leaves of the C3 plant act as a source for sucrose synthesis, which is transported to the sink organs to support plant growth [13]. During the day, photosynthetic CO_2_ fixation drives the synthesis of sucrose and starch accumulation, which are used to facilitate continued sucrose production at night [13,14]. At night, starch stored during the day is degraded and consumed until dawn. Under a wide range of day-night lengths, the rate of starch accumulation and degradation in Arabidopsis leaves are essentially linear and approximately 95% of the starch is used with dawn [1,14,15]. Disturbances of starch turnover by unexpected early onset of nighttime lead to premature exhaustion of starch and carbon starvation [14]. This carbon starvation can result in rapid changes in metabolism and gene expression and small changes in growth rate, which can lead to much larger changes in biomass production within 2–3 weeks [1,16,17]. It is possible to directly or indirectly control these mechanisms of the biological clock genes.

Transcript abundance of many starch genes is regulated by the circadian clock [18,19]. For example, *granule-bound starch synthase 1* (*GBSS1*) gene expression in Arabidopsis leaves is controlled by Myb-related *circadian clock associated 1* (*CCA1*) and *late elongated hypocotyl* (*LHY*) clock transcription factors [20], and the *ADP-glucose pyrophosphorylase* (*AGPase*) gene exhibits a circadian rhythm with a different diurnal fluctuation pattern from *GBSS1* [21]. Regulation of the starch *GBSS1* gene in sweet potato leaves by the circadian clock was also reported by Wang et al. [19]. In tobacco, circadian oscillations have been reported for glutamate synthase and glutamate dehydrogenase activity, and the nitrate reductase RNA level and enzyme activity has been found to change within 24 h cycles [2]. In cotton seedlings, the circadian cycle has been found to be associated with lipid biosynthesis [22]. In that study, the levels of linoleic and linolenic acids fluctuated with higher amounts in the middle of the night than in the middle of the day and these oscillations continued under constant light conditions. Growth rates of sink organs also depend on the 24-h cycle. In potato leaves, starch is synthesized and degraded diurnally, and in potato tubers, starch is accumulated and stored for a long time. Expression of sucrose synthase and AGPase genes is closely related to starch metabolism and follows the diurnal rhythm in leaves and tubers [23,24]. The relationship between starch metabolism and degradation depends on circadian rhythm and tuberization, but is still largely elusive in potato. Starch metabolism and use is under circadian regulation. CO_2_ assimilation and starch and sugar concentrations oscillate in continuous light after entrainment [1,9,25].

The rate of CO_2_ assimilation oscillates in wild-type seedlings under continuous light following entrainment but is arrhythmic in CCA1-ox plants. In addition, CCA1-ox plants had lower chlorophyll content, reduced CO_2_ assimilation, and reduced biomass compared to the wild type [9]. Experiments using *gi* mutants in Arabidopsis and rice [25] suggested that *GIGANTEA* (GI) plays an important role in controlling the cycle of glucose metabolism in plants since the amplitude is elevated under endogenous sucrose and starch concentrations. In Arabidopsis leaves, circadian clocks control starch degradation rates for optimal carbon assimilation, suggesting that they are important for increasing crop productivity and for plant growth and development [15].

## 3. Regulation of Starch Metabolites by the Circadian Clock

The abundance of evidence for the diurnal behavior of the primary metabolite pathway indicates that the manipulation of carbon metabolites increases crop productivity. Starch and sugar metabolism are an important circadian output contributing to optimization of plant physiology. We can try to improve productivity of crop plants through circadian-mediated up-regulation of photosynthetic carbon assimilation. To accomplish this, we will need to focus on circadian clock genes related to the regulation of starch accumulation and degradation.

In higher plants, many reports have shown that the circadian rhythm plays a major role in several steps in coordinating metabolic pathways associated with carbon fixation and allocation between starch and sucrose in leaf tissue [10,21,26,27]. During the day, triose-phosphates fixed by photosynthesis are partitioned to synthesize sucrose and starch. The key enzymes of the sucrose synthesis pathway, fructose 1,6-bisphosphatase (cFBPase) and sucrose phosphate synthetase(SPS), are inhibited by SnRK1 kinase (SNF1-related kinase 1), which is activated by its b subunit AKINb1 [28,29,30]. Sucrose synthesis is also activated by osmo-sensitive kinase OsmK, which senses rhythmic changes in water deficit via Ca^2+^-dependent kinase (CaK) [31,32]. Both diurnal sensors b and CaK are regulated by light and the circadian clock. The b subunit is up-regulated by darkness and the circadian protein LHY [33] and CaK are activated by light and inhibited by LHY [13]. At night, OsmK accelerates starch degradation to up-regulate sucrose production through the positive interactions between source supply and sink demand, which are gated by the clock via CaK [13,31].

**Figure 1:**
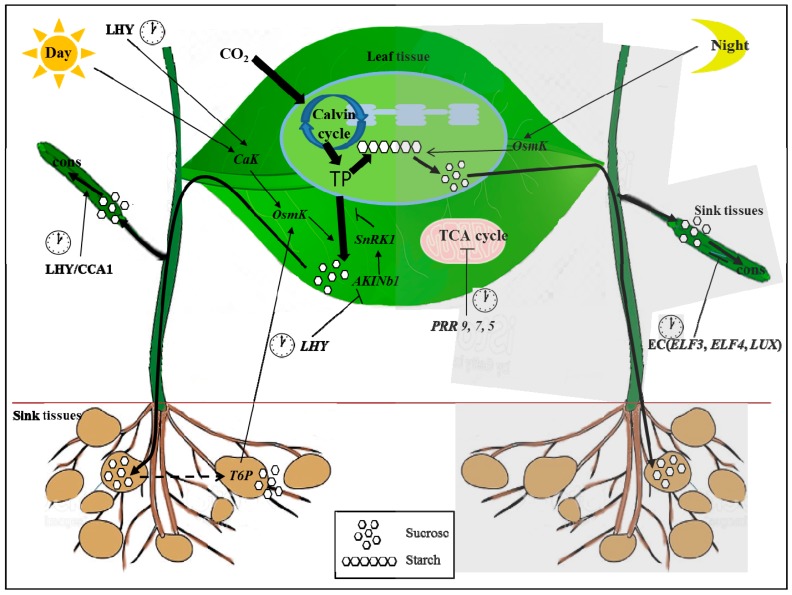
A schematic illustration of the relationship between the circadian clock and carbohydrate metabolism. Information from the circadian clock is transmitted to the chloroplast and mitochondria. Triose-phosphates (TP) fixed during the day by photosynthesis are partitioned to synthesize sucrose and starch. During the day, sucrose synthesis is inhibited by the *SNF1-related kinase 1* (*SnRK1*) and is activated by the *osmo-sensitive kinase OsmK* [13]. *SnRK1* and *OsmK* sense rhythmic changes by light and the clock protein late elongated hypocotyl (*LHY*). Sucrose is exported and consumed by sink tissues. *Trehalose 6-phosphate* (*T6P*) accelerates the development of sink tissues, thus increasing the sink demand for carbon during the day. Increased demand in turn activates *OsmK*, creating a positive feedback loop activating the source supply by sink demand. At night, *OsmK* accelerates starch degradation and thus up-regulates sucrose production. Therefore, activation of the sucrose supply during the day increases the sink demand, which in turn increases *OsmK* and up-regulates starch degradation and thus sucrose supply at night. Consumption of sugars by sink tissues is regulated to the clock via activation by *LHY/CCA1* and inhibition by the evening complex (EC) (*Early Flowering 3* (*ELF3*), *ELF4*, and *lux arrhythmo* (*LUX*)). The clock genes *pseudo-response regulator 5* (*PRR5*), *7*, and *9* regulate the tricarboxylic acid cycle (TCA) at night [43,44]. Black thick lines on plants and show carbon movement. Arrow end lines and blocked end lines indicate activate and inhibit the reactions and expressions, respectively. Night reactions are written in gray box. Clock cartoons emphasize the clock genes. Arrow end dotted line indicates feedback control of sugar by sink strength [13]. The character of cons means consumption of sugar.

Two other key transcriptional activators of plant growth, phytochrome interacting factor 4 (PIF4) and PIF5, regulate consumption of sugars through the circadian clock [34]. These transcription factors are activated by the morning clock proteins LHY/CCA1 and are inhibited by the evening complex (EC), Early Flowering 3 (ELF3), ELF4, and lux arrhythmo (LUX) [34,35]. Thus, the LHY/CCA1 complex contributes to the regulation of carbon partitioning (through b) and starch degradation (through CaK) and the EC only regulates consumption [13] (Figure 1).

When Arabidopsis is grown under abnormal day lengths (17 or 28 h), starch was exhausted until exposure to light, irrespective of the actual dawn. In the short-period mutant *cca1-11 lhy-21* (17 h) [36,37], starch degradation was faster than the wild type under normal, 24-h LD (Long Day; 16 h day and 8 h night) cycles [1]. While the *cca1-11 lhy-21* mutant plants grown in 17-h LD cycles show a starch degradation pattern similar to wild-type plants grown in normal 24-h LD cycles, wild-type plants grown in 28-h LD cycles and *cca1 lhy* mutant plants grown in 24-h LD cycles show symptoms of carbon starvation for the last few hours of the night [1]. This ‘early dusk’ starvation affects plant growth. Under the same amount of light and rate of photosynthesis, growth of wild-type Arabidopsis is reduced by a third or more in 28-h LD cycles relative to 24-h LD cycles [1]. Mobilization of starch reserves by matching the length of the LD cycles and the clock period is essential for optimal biomass accumulation [38]. The Arabidopsis *elf3* mutant [6] displays a slightly slower starch degradation rate than the wild type [13,39]. *ELF3* plays a role in sustaining the rhythm by inhibiting phototransduction at dusk [40], and inhibits hypocotyl growth in the light and maintains this inhibition during night. However, *elf3* mutants immediately lose circadian rhythms [41] and show relieved inhibition of hypocotyl growth early in the night [42]. In addition, the *elf3* mutant shows a two-fold higher root growth rate in the light period and two-fold inhibition during the night [39]. The missing inhibition of consumption by the EC at night perturbed the balance between the sugar supply and consumption and consequentially activated the starvation mechanisms [13].

In addition, a dramatic increase of intermediates in the tricarboxylic acid cycle (TCA) in a triple mutant of *pseudo-response regulators* (*PRR 9*, 7, and *5*) showed that these proteins are involved in maintaining mitochondrial homeostasis in Arabidopsis [43]. By analyzing the transcriptome and metabolome, we assumed that PRR 9, 7, and 5 negatively regulate the biosynthetic pathways of photorespiration, chlorophyll, carotenoid, and the antioxidant compounds, abscisic acid and α-tocopherol [43] (Figure 1).

Clock genes match the endogenous clock period with the period of exogenous LD cycles and can ultimately maximize plant growth and metabolites by optimizing the phase relation between clock-controlled biology and exogenous day-night cycles [8,9]. On the other hand, transgenic potato plants altered the diurnal rhythm of carbon allocation patterns through antisense inhibition of triose-phosphate phosphate translocator (TPT), resulting in reduced starch production and consequently a change in the diurnal growth pattern [45]. Espinoza et al. [9] reported that the circadian clock function was disrupted under low temperature stress conditions and, therefore, cycles of many clock components and output genes were disrupted. During cold acclimation, some enzymes and genes related to starch catabolism in chloroplasts showed reduced amplitude in the diurnal oscillations or arrhythmic expression patterns [11]. The study between clock and circadian regulation of metabolism will help improve crop productivity by reflecting the sensitization of plants to environmental inputs [2,46].

## 4. Regulation of Circadian-Mediated Carbon Productivity in Crops

When the circadian clock is correctly matched with environmental periods the photosynthetic capacity and carbohydrate metabolism are enhanced [9,15,47,48,49,50]. Manipulating the circadian clock in crop plants may help to overcome problems of climate change and food deficiency. However, translating molecular mechanisms of model plants to crop plants is difficult because many crop plants of agricultural significance have experienced diverse evolution (Table 1) [51,52,53,54,55].

In the rice plant, a model plant of monocotyledon, many experiments have induced mutations in circadian-related genes. In *OsGIGANTEA* (*OsGI*), the rice circadian clock-related null mutant, several clock related genes show changes in gene expression or rhythm. In the *osgi* mutant, *OsLHY* (clear rhythm, reduced expression), *OsPRR1* (reduced rhythm, up-regulated expression level), *OsPRR95*, and *OsPRR59* (reduced rhythm, similar level of expression) showed changes in expression under the natural day-night conditions in the field [56,57].

The expression of 75% of 27,201 tested genes, including *LHY*-related genes, *LUX*, *ELF3*-like, and several starch synthesis-related genes, was affected in the *osgi* mutant. These changes in the transcriptome resulted in increased sucrose and starch content, reduced chlorophyll content, and increased stomatal conductance in the leaves of *osgi* mutants [57]. Moreover, the production of malate, citrate, 2-oxoglutarate, aconitate, and isocitrate in the TCA cycle were significantly increased or decreased. However, these changes do not result in changes to related metabolites and do not affect the photosynthetic rate in the field [57]. Rather than the TCA cycle itself, the changes in the pool sizes of some transcripts and these primary metabolites lead to activation of secondary metabolism, such as the phenylpropanoid metabolite pathway [57].

In *Brassica rapa*, three crop morphotypes, called leaf, turnip, and oilseed, have evolved differently as a result of crop diversification after domestication in diverse geographic regions [58,59]. Vegetable and oilseed varieties have dramatically different morphology and harvestable components [60]. Among these varieties, the circadian period, gas exchange, and physiological traits are also different. Circadian period is positively correlated with the maximum net photosynthesis rate but is negatively correlated with stomatal conductance [61]. The oilseed crop has a shorter circadian period (closer to 24 h) and higher net carbon assimilation and stomatal conductance relative to the vegetable types. Biomass is also positively associated with the circadian period. While the maximum net photosynthesis rate of leaf and turnip crops is related to the mass or allocation of fixed carbon in vegetative organs, the metabolically expensive seed oil affects the total biomass accumulation in oilseed crops [61]. 

In potato, the circadian clock gene that perceives day length is important for controlling potato tuberization. The *Solanum tuberosum* constans (StCO) protein, which is regulated by the circadian clock gene, is involved in the photoperiodic control of flowering and tuberization. StCO is involved in day–length control of tuberization, where it represses tuber formation in non-inductive long days by inhibiting the mobile tuberizing signal, called *StSP6A*, in the leaves [62]. During short days, the circadian clock gene does not form the *StGI1/StFKF1/stCDF1* complex, thus inhibiting expression of the *StCO* gene. Therefore, the phloem-mobile *StSP6A* mRNA, which promotes potato tuberization, is transferred to the stolon from the leaves. It seems that the circadian rhythm is closely related to genes involved in tuber formation in potatoes. 

## 5. Conclusions

Plants respond to environmental changes by triggering biochemical and developmental networks [38]. As a result of the rotation of the earth on its axis, the circadian clock provides plants the ability to adapt to daily changes in environmental conditions and the ability to time the production and consumption of energy. This circadian clock controls many developmental processes, which are related to the primary metabolite pathway, throughout the entire life cycle of the plant. In addition, the circadian system plays a role in regulating responses to both biotic and abiotic stresses [66]. Therefore, understanding the relationship between plant metabolic pathways and clock mechanisms improves biological understanding and yield prediction. In particular, crops that have experienced genome duplication through evolution and domestication have diverse gene families and unpredictable relationships between molecular changes in the field [57,61,67]. The morphology of the crop is an important characteristic compared with the model plant, Arabidopsis. Certain organs are hypertrophic or have a specific mechanism that induces the development of organs, and the production of metabolites may occur at specific times. Human edible parts also depend on the shape and developmental stage of the crop. The type of crop is determined by the intensive allocation of the crops due to the accumulation of carbohydrate metabolites, and the size and metabolite accumulation of these edible parts have been evolutionally increased by human selection [61,68,69]. Recent studies have revealed that the circadian clock regulates starch utilization and photosynthesis [1,9,70] and photosynthetic evolution alters sugar status and thereby affects clock function [71,72]. The circadian clock directly regulates metabolism related to plant development, growth, and metabolite products and also affects crop productivity and quality through metabolite changes in response to abiotic stress [11,43,73,74]. These findings suggest that manipulation of circadian clock genes can be valuable for improvement of plant crop yield.

## Figures and Tables

**Table 1:** Clock-related genes reported in the references.

Name of Gene	Arabidopsis	Homologus Genes in the Crops	Reference
**Clock genes**			
***Late elongated hypocotyl* (*LHY*)**	AT1G01060	*OS-LHY* (*Oryza sativa*, rice) *LHY* (*Solanum tuberosum, potato*)	Izawa et al., 2011 [57] Hancock et al., 2014 [44]
***Circadian clock associated 1* (*CCA1*)**	AT2G46830	N.A.*	
***Early Flowering 3* (*ELF3*)**	AT2G25930	*OsELF3* (*Oryza sativa*, rice)	Kwon et al., 2015 [35]
***Early Flowering 4* (*ELF4*)**	AT2G40080	*OsELF4* (*Oryza sativa*, rice)	
***Lux arrhythmo* (*LUX*)**	AT3G46640	*OsLUX* (*Oryza sativa*, rice)	Kwon et al., 2015 [35]
***Pseudo-response regulator* (*PRR*)*s***	AT1G32100 AT5G60100 AT5G24470 AT5G02810 AT2G46790	*OsPRRs* (*Oryza sativa*, rice) *BrPRRs* (*Brassica rapa,* Chinese cabbage)	Murakami et al., 2003 [56] Kim et al., 2007, 2012 [63,64]
***GIGANTEA* (*GI*)**	AT1G22770	*OsGI* (*Oryza sativa*, rice) *StGIa*,*b* (*Solanum tuberosum, potato*) *GmGIa* (*Glycine max, soybean*) *IbGI* (*Ipomoea batatas Lam, sweet potato*) *BoGI* (*Barssica oleracea L. broccoli*) *BrGI* (*Brassica rapa, Chinese cabbage*) *LpGI* (*Lolium perenne, ryegrass*)	Kwon et al., 2015 [35] Hancock et al., 2014 [44] Watanabe et al., 2011 [55] Tang et al., 2017 [54] Thiruvengadam et al., 2015 [53] Kim et al., 2016 [52] Gagic et al., 2015 [51]
**Clock sensing genes**			
***Ca^2+^-dependent kinase* (*CaK*)**		N.A.	
***SNF1-related protein kinase 1* (*SnRK1*)**	AT5G39440	*SnRK1* (*Spinacia oleracea, spinach*) *SnRK1* (*Nicotiana tabacum, tobacco*) *SnRK1* (*Secale cereale, rye*) *SnRK1* (*Solanum tuberosum, potato*) *SnRK1* (*Hordeum vulgare barley*) *SnRK1* (*Triticum aestivum, wheat*)	Sugden et al., 1999 [29] Sugden et al., 1999 [29] Sugden et al., 1999 [29] Sugden et al., 1999 [29] Halford et al., 2003 [28] Coello et al., 2012 [65]
***ARABIDOPSIS KINASE* (*AKIN*) *b1***	AT5G21170	N.A.	
***osmo-sensitive kinase (OsmK)***	N.A		

* N.A. Not Available.
